# A comparative study of Taiwan's short-term medical missions to the South Pacific and Central America

**DOI:** 10.1186/1472-698X-12-37

**Published:** 2012-12-27

**Authors:** Ya-Wen Chiu, Yi-Hao Weng, Chih-Fu Chen, Chun-Yuh Yang, Hung-Yi Chiou, Ming-Liang Lee

**Affiliations:** 1 Division of Preventive Medicine and Health Services Research, Institute of Population Health Sciences, National Health Research Institutes, Miaoli, Taiwan; 2 Master Program in Global Health and Development, College of Public Health and Nutrition, Taipei Medical University, Taipei, Taiwan; 3 Department of Pediatrics, Chang Gung Memorial Hospital, Chang Gung University College of Medicine, Taoyuan, Taiwan; 4 International Cooperation and Development Fund, Taipei, Taiwan; 5 Department of Public Health, Kaohsiung Medical University, Kaohsiung, Taiwan; 6 School of Public Health, College of Public Health and Nutrition, Taipei Medical University, Taipei, Taiwan; 7 Taiwan Health Corps, Taipei, Taiwan

**Keywords:** Medical missions, Health professional, Language, Education, Efficiency

## Abstract

**Background:**

Taiwan has been dispatching an increasing number of short-term medical missions (STMMs) to its allied nations to provide humanitarian health care; however, overall evaluations to help policy makers strengthen the impact of such missions are lacking. Our primary objective is to identify useful strategies by comparing STMMs to the South Pacific and Central America.

**Methods:**

The data for the evaluation come from two main sources: the official reports of 46 missions to 11 countries in Central America and 25 missions to 8 countries in the South Pacific, and questionnaires completed by health professionals who had participated in the above missions. In Central America, STMMs were staffed by volunteer health professionals from multiple institutions. In the South Pacific, STMMs were staffed by volunteer health professionals from single institutions.

**Results:**

In comparison to STMMs to Central America, STMMs to the South Pacific accomplished more educational training for local health providers, including providing heath-care knowledge and skills (p<0.05), and training in equipment administration (p<0.001) and drug administration (p<0.005). In addition, language constraints were more common among missions to Central America (p<0.001). There was no significant difference in the performance of clinical service between the two regions.

**Conclusions:**

Health-care services provided by personnel from multiple institutions are as efficient as those from single institutions. Proficiency in the native language and provision of education for local health-care workers are essential for conducting a successful STMM. Our data provide implications for integrating evidence into the deployment of STMMs.

## Background

Medical missions are a means of extending the reach of healthcare professionals to the underdeveloped world. A number of organizations and countries provide medical care through such initiatives [1-5]. Such endeavors improve health for people worldwide and reduce the health disparity among populations. They can have a significant impact on not only patients but also the healthcare workers themselves [6,7]. The success of these efforts has the additional benefit of helping improve the willingness of health professionals to volunteer to serve in underdeveloped locations [8]. Medical missions are categorized into short- and long-term operations. Over the years, as many long-term healthcare operations have been replaced by sector-wide approaches, there has been a shift in focus from long-term overseas assignments to short-term medical missions (STMMs) [9]. There are three types of STMMs: emergency, surgical, and mobile services. Emergency services provide post-disaster medical relief care anywhere in the world. Surgical services provide dental and surgical aid in regions where such services are generally unavailable [7]. Mobile services provide clinics in outlying villages where medical care is either lacking or inadequate.

Concerns have been raised regarding the long-term impact of STMMs on recipient communities [10-12]. Constraints against such missions include a lack of follow-up care, short duration, too many patients, linguistic and cultural barriers, limited medications and supplies, and a lack of support services from local authorities. An understanding of partnerships between the humanitarian organization and its host country is beneficial to reduce the risks to aid workers and the potential harm to recipients [13]. Therefore, a standardized deployment strategy is required to assure their quality [14,15].

A variety of healthcare professionals have engaged in providing humanitarian aid through STMMs for decades; but few studies have examined their perceptions [4,11,12,16,17]. Quantitative studies investigating the efficiency of STMMs could help improve the implementation of such missions. In an attempt to determine helpful strategies for the deployment of STMMs, we conducted a cohort survey to analyze Taiwan’s STMMs dispatched to the South Pacific and Central America. We have developed a profile of STMMs and identified important characteristics worthy of further attention.

### Taiwan’s STMMs

The Taiwan International Cooperation and Development Fund (TaiwanICDF) is the principal official body superintending Taiwan’s overseas cooperative programs. Since 2005, the TaiwanICDF has coordinated with 37 hospitals to dispatch groups of health professionals, in the form of STMMs, to Taiwan’s partner countries. Therefore, many health professionals have been called upon to provide short-term medical care to patients in villages in underdeveloped countries. The dispatch of STMMs is to improve the healthcare environments of Taiwan’s allies by offering education and training, as well as guidance, experience, and methodologies regarding disease control as practiced in Taiwan. These efforts involve groups of healthcare personnel traveling to medically underserved areas far from Taiwan.

### Preparedness of STMMs

Taiwan’s allies include 11 countries in Central America (Guatemala, Honduras, Haiti, Saint Lucia, Nicaragua, Panama, El Salvador, Belize, Dominican Republic, Paraguay, and Saint Kitts and Nevis) and 8 countries in the South Pacific (Kiribati, Papua New Guinea, Tuvalu, Palau, the Solomon Islands, the Marshall Islands, Fiji, and Nauru). The official language is Spanish in most of these countries in Central America (63.6%), including Guatemala, Honduras, Nicaragua, Panama, El Salvador, Dominican Republic, and Paraguay. In contrast, the official language in all of these South Pacific countries is English (100%).

Diplomatic officials with the Ministry of Foreign Affairs (MOFA) of the Republic of China (Taiwan) arrange meetings with medical officers in their host nation whenever STMMs are requested. Surveys are conducted to gather relevant information, including the how long the mission would last, what specialties mission personnel might need, the locations of mobile clinics, licensure of medical care, and issues relating to the import of drugs and equipment.

### Recruitment of STMM members

Once a mission is confirmed, the TaiwanICDF recruits health professionals from cooperating hospitals. STMMs comprise a team of specialist physicians (mainly internists, surgeons, dentists, and pediatricians), experienced nurses, pharmacists, and public health personnel. Fluency in English is required. All members receive disease prophylaxis, such as vaccinations, prior to departure [18].

The Taiwanese government encourages Taiwanese medical institutions to cooperate with medical institutions of allies in the South Pacific by providing short-term clinical services and technical support. Agreement has also been reached to formulate a Taiwan-Pacific Medical Alliance. Table 1 summarizes the relationships between sister hospitals in Taiwan and the South Pacific. Overall, six Taiwanese medical institutions are twinned with eight South Pacific hospitals, with each Taiwanese medical institution recruiting team members from within its own institute for deployment to host nations. In contrast, each STMM dispatched to Central America is staffed by volunteer health professionals from multiple institutions.

**Table 1:** Sister hospitals for STMMs to the South Pacific

**Country**	**Sister hospital**	**Sister hospital in Taiwan**
Kiribati	Tungaru Central Hospital	Mackay Memorial Hospital
Tuvalu	Princess Margaret Hospital	Chung Shan Medical University Hospital
Palau	Palau National Hospital	Wan Fang Hospital
Solomon Islands	Honiara Central Referral Hospital	Kaohsiung Medical University Chung-Ho Memorial Hospital
Marshall Islands	Majuro Hospital	Wan Fang Hospital
Nauru	Nauru General Hospital	Show Chwan Memorial Hospital
Fiji	Colonial War Memorial Hospital	Mackay Memorial Hospital
Papua New Guinea	Wewak Boram hospital	Changhua Christian Hospital

A comparison of the preparedness of STMMs to the South Pacific and to Central America is summarized in Table 2. The most significant difference between the two regions was the single- or multiple-institution nature of mission recruitment and the presence or absence of sister hospitals in host nations. There was no difference in the funding sources, leadership, administration, logistical preparedness, or orientation between the two regions.

**Table 2:** Preparedness of STMMs dispatched to the South Pacific and Central America

** Region**	**South pacific number of missions** = **46**	**Central America number of missions** = **25**
Funding source	MOFA, TaiwanICDF	MOFA, TaiwanICDF
Mission leader	TaiwanICDF	TaiwanICDF
Administrative personnel	TaiwanICDF	TaiwanICDF
Logistical preparedness	TaiwanICDF	TaiwanICDF
Orientation	TaiwanICDF	TaiwanICDF
Member recruitment	Single institutions	Multiple institutions
Doctors	Specialty	Specialty
Nurses	Senior	Senior
Other	Experienced	Experienced
Sister hospital in host nation	Yes	No

### Logistics and orientation of STMMs

MOFA and the TaiwanICDF sponsor all costs associated with STMMs, including transportation and travel, daily expenses, logistical support, medication and supplies, laboratory capabilities, and accommodation. Drugs and equipment are prepared by participating hospitals in accordance with guidelines published by the World Health Organization and the International Dispensary Association [6]. No medications are obtained locally.

The TaiwanICDF arranges a two-hour orientation for all participants one to two weeks before each STMM is dispatched. The content of this program of orientation includes an introduction to the host nation and its medical problems, a mission schedule, and information on the duties of STMM members. Each participant receives a booklet about the current mission and travel destination. The quality of each orientation is similar.

### Medical services

Once members of STMMs have arrived in the field, diplomats from Taiwan’s Ministry of Foreign Affairs arrange local transportation and accommodation for them. Those on STMMs are instructed to minimize any disruption to the culture of their host countries, conform to levels of local health care, and facilitate cooperation with local healthcare workers, including medical students.

## Methods

### Study population

The targets were members of STMMs dispatched by TaiwanICDF to the South Pacific and Central America during 2005 to 2010.

### Daily reports of STMMs

During the mission period, STMM members complete daily work records, which are collated, compiled, and included in the single-volume final report that documents the work of each STMM. In this study, all reports (a total of 71 volumes) from 2005 to 2010 were collected for review. Data retrieved included locations, mission duration, mission size, and patient numbers. Educational materials provided by members of STMMs were also collected. The missions to the South Pacific and Central America were then analyzed, especially for any differences.

### Questionnaire survey for members of STMMs

A postal questionnaire survey was provided to all STMM members participating in the missions dispatched by TaiwanICDF to the South Pacific and Central America during 2005 to 2010. The development of the questionnaire to assess STMMs was based on a comprehensive literature review of publications of existing questionnaires [5,14]. In this survey, the content validity index of 0.94 and Cronbach’s coefficient alpha of 0.73 indicate sufficient validity and reliability of parameters in the questionnaire.

The questionnaire was anonymously distributed to all members of 71 STMMs in a four-month period from January through April 2011. An introductory letter that stated the purpose of this study and assured confidentiality was accompanied. The study protocol was approved by the Research Ethics Committee of the National Health Research Institutes, Taiwan.

The questionnaire consisted of items for measuring the perceptions and efficiency of STMMs. The efficiency portion covered orientation efforts, intra-team communication, utilization of time, educational training of local health workers, necessary personnel, health care, and interpreter services. Questions regarding efficiency of STMMs were rated by Likert’s 5-point scale (*completely agree*, *agree*, *neutral*, *disagree*, *completely disagree*). In addition, questions about proficiency in the language of the destination country and knowledge of local culture were asked. Furthermore, background characteristics — including gender, age, marital status, academic degree, profession, and work experience — were also explored.

### Statistical analyses

Statistical analyses were conducted using a commercially available program (SPSS 12.0 for Windows, SPSS Inc., Illinois, USA). Likert’s 5-point scale was dichotomized for further analyses. A self-rating report of either *agree* or *completely agree* was regarded as a favorable answer. The other three (*neutral*, *disagree*, and *completely disagree*) were viewed as unfavorable perceptions. Categorical variables were analyzed using the chi-square test or Fisher’s exact test. For comparison between groups with quantitative variables, the null hypothesis that there was no difference between each group was tested by ANOVA. Significance was defined as *p* < 0.05.

## Results

### Taiwanese STMMs

During the six-year study period, the TaiwanICDF organized 71 medical missions to 19 countries, including 25 missions to 11 nations in Central America and 46 missions to 8 nations in the South Pacific. Table 3 summarizes the details of these missions. The length of each mission ranged from 5 to 14 working days, while the number of members per mission ranged from 5 to 22 persons, the number of patients per mission ranged from 15 to 4,659 persons, and the number of locations per mission ranged from 1 to 10.

**Table 3:** Structure of STMMs dispatched to the South Pacific and Central America

**Country**	**STMM number**	**Size** (**Personnel**)	**Working days**	**Number of service locations**	**Patients**
**Central America**, **in total**	**25**	**271**	**231**	**107**	**52179**
Guatemala	5	10–12	10–13	4–10	3046–4659
Honduras	3	10–12	7–10	2–4	1374–2629
Haiti	3	9–15	8–12	1–4	183–4224
Saint Lucia	1	10	6	5	252
Nicaragua	2	11–14	8–10	2–4	2600–2661
Panama	2	8–10	9–10	5–10	1566–1566
El Salvador	2	11–13	10	4	3083–3513
Belize	1	11	9	6	739
Dominican	1	15	7	3	890
Paraguay	3	7–11	5–11	1–4	15–1186
Saint Kitts and Nevis	2	9	8–9	3–4	184–441
**South Pacific**, **in total**	**46**	**417**	**385**	**124**	**32708**
Kiribati	8	6–11	5–9	1	300–787
Papua New Guinea	2	10–22	9	4–7	416–668
Tuvalu	8	5–10	7–10	1–4	395–912
Palau	4	7–12	5–14	1–6	300–864
Solomon Islands	5	8–18	6–10	5–9	340–2393
Marshall Islands	9	7–13	6–13	1–4	294–996
Fiji	3	7–12	6–8	4–6	300–1199
Nauru	7	6–11	5–10	1–2	476–1141

### STMM services

The services provided by STMMs are summarized in Table 4. For both the South Pacific and Central America, STMM’s main health professionals were medical doctors. Pharmacists were not always enrolled in STMMs. There were significant structural differences in the STMMs — including the number of mission members (p < 0.01), the number of patients (p < 0.001), and location (p < 0.01). In Central America, the most common locations visited were communities; in the South Pacific, hospitals. Missions to the South Pacific more often provided educational training (such as lectures, written media, and hands-on training) — including knowledge and skills in health care (p < 0.05), equipment administration (such as maintenance of medical equipment) (p < 0.001), and drug administration (such as usage of medications) (p < 0.005) — to local health professionals than those in Central America.

**Table 4:** Services provided by 46 STMMs to the South Pacific and 25 STMMs to Central America

** Region**	**South Pacific N** =**46**	**Central America N** =**25**	***P *value**
**Size** (**person**/**mission**)	9.07 ± 0.46	10.84 ± 0.41	< 0.01
**Primary care professionals**	7.43 ± 0.36	9.24 ± 0.39	< 0.005
Doctors	5.35 ± 0.24	6.12 ± 0.31	
Nurses	1.59 ± 0.13	2.16 ± 0.07	
Pharmacists	0.49 ± 0.08	0.96 ± 0.09	
**Working day** (**days**/**mission**)	8.37 ± 0.28	9.24 ± 0.38	0.0685
**Patient** (**patients**/**mission**)	711 ± 54	2087 ± 314	< 0.001
**Location** (**locations**/**mission**)	2.70 ± 0.32	4.28 ± 0.48	< 0.01
Hospital	1.31 ± 0.09	1.40 ± 0.24	
Primary care center	0.72 ± 0.20	1.24 ± 0.34	
Community (e.g. school)	0.67 ± 0.15	1.64 ± 0.52	
**Education** (%)			
For **health workers**			
Knowledge and skills in health care	46 (100.0)	21 (84)	< 0.05
Equipment administration	34 (73.9)	7 (28)	< 0.001
Drug administration	23 (50.0)	3 (12)	< 0.005
**For the general population**			
Public health	46 (100.0)	23 (92)	0.12

### Perceptions and efficiency of STMMs

Questionnaires were mailed to 383 health professionals who had participated in STMMs dispatched by TaiwanICDF to the South Pacific or Central America. Among them, 253 questionnaires (66.1%) were valid for analyses, including 153 from missions dispatched to the South Pacific and 100 from missions dispatched to Central America. Their personal characteristics are provided in Table 5. There was no significant difference in the demographic data between personnel of the South Pacific and Central America — including in the areas of gender, age, marital status, working experience, academic degree, and profession.

**Table 5:** Demographic data of 253 health professionals participating in STMMs

** Region**	**South Pacific N** = **153**	**Central America N** = **100**	***P *value**
**Age** (**years**)	41.0 ± 8.6	40.9 ± 10.5	0.876
**Working period** (**years**)	13.7 ± 8.4	13.9 ± 9.3	0.866
**Gender** (**male**, %)	82 (53.6)	50 (50.0)	0.576
**Married** (%)	81 (52.9)	45 (45.0)	0.217
**Academic degree** (%)			0.057
High school or below	13 (8.5)	4 (4.0)	
College*	79 (51.6)	68 (68.0)	
Master’s	48 (31.4)	20 (20.0)	
Ph.D.	13 (8.5)	8 (8.0)	
**Profession**			0.994
Doctor	79 (51.6)	51 (51.0)	
Nurse	45 (29.4)	30 (30.0)	
Allied health profession	29 (19.0)	19 (19.0)	

* College curriculum is 7 years for medical school, 6 year for dental school, and 4 years for the other specialties.

Health professionals’ perceptions and efficiency of STMMs are summarized in Table 6. Frequently cited reasons for their engagement in STMMs included personal development (78.7%), providing health care (76.3%), and helping Taiwan’s diplomatic relations (72.7%). Less than half of participants rated training for local health providers as an important goal (46.6%). There were no significant differences in the expectations of participation in STMMs between health professionals in missions in the South Pacific and those in Central America. Furthermore, health personnel in missions of the South Pacific perceived a better ability to speak the local language than those in Central America (p < 0.001). They less often required interpreters for clinical practice (p < 0.001). There were no significant differences in the following perceptions between STMMs of the South Pacific and those of Central America: knowledge of local culture, orientation efforts, intra-team communication, utilization of time, and necessary personnel.

**Table 6:** Perceptions of 253 health professionals toward STMMs

**Perception**	**South Pacific N** = **153**	**Central America N** = **100**	***P *value**
**Expectation**			
Health care	113 (73.9)	80 (80.0)	0.261
Personal development	123 (80.4)	76 (76.0)	0.405
Enhance Taiwan’s diplomatic relationship	109 (71.2)	75 (75.0)	0.512
Training for local health providers	69 (45.1)	49 (49.0)	0.543
**Belief**			
Language proficiency enhances efficiency	129 (84.3)	85 (85.0)	0.883
Cultural awareness enhances care quality	139 (90.8)	89 (89.0)	0.630
Education helps local health providers to improve clinical care	137 (89.5)	87 (87.0)	0.602
Subsequent follow-up improves care outcome	142 (38.6)	91 (91.0)	0.535
**Requirement of interpreter service**	104 (68.0)	97 (97.0)	<0.001
**Ability to speak the native language**			<0.001
None	17 (11.1)	50 (50.0)	
Basic	56 (36.6)	37 (37.0)	
Conversational	59 (38.6)	9 (9.0)	
Proficient	13 (8.5)	3 (3.0)	
Fluent	8 (5.2)	1 (1.0)	
**Knowledge of local culture**			0.306
None	18 (11.8)	14 (14.0)	
A little	46 (30.1)	38 (38.0)	
Average	70 (45.7)	42 (42.0)	
Know well	18 (11.8)	6 (6.0)	
Extremely familiar with	1 (0.6)	0 (0.0)	
**Efficiency** (%)			
Orientation efforts were well organized	99 (64.7)	62 (62.0)	0.662
Intra-team communication was efficient	121 (79.1)	74 (74.0)	0.347
Time was spent efficiently	122 (79.7)	82 (82.0)	0.656
Health care services were satisfied	129 (84.3)	86 (86.0)	0.714
Necessary personnel were sufficient	36 (23.5)	25 (25.0)	0.789
Interpreter services were adequate	150 (98.0)	100 (100.0)	0.159
Education of local health providers was well organized	80 (52.3)	44 (44.0)	0.197

## Discussion

This study represents the first large-scale cohort analysis to address Taiwan’s provision of STMMs. We have identified the scope and content of Taiwan’s STMMs sponsored by the TaiwanICDF. Our results demonstrate that most participants were satisfied with intra-team communication, interpreter services, and healthcare services. These findings suggest that STMMs established by the TaiwanICDF are well planned and efficient in providing health care to host nations. We found five main common characteristics among Taiwan’s STMMs. First, the TaiwanICDF collaborated with 37 hospitals to offer the medical contingent from surge capacity. Second, the health logistics are delivered from Taiwan. Third, STMM personnel are mostly physicians. Fourth, local inhabitants, including expatriates, made up part of the healthcare team through collaborative effort (data not shown). And fifth, the missions are mobile; with most serving multiple locations.

STMMs can provide unique experiences for their participants. In developed countries, many institutes arrange electives to participate in health care overseas [2,8]. They provide global health education to broaden physicians’ own clinical practices and enhance their humanitarian efforts. The success of STMMs has the benefit of helping improve the willingness of health professionals to volunteer to serve in underdeveloped locations. Other than the short orientation STMM members receive, there is no training program in Taiwan yet for medical missions.

In our study, approximately three-fourths of participants rated STMMs as a means to gain and maintain diplomatic friendships. In contrast to religious and faith-based organizations [19,20], Taiwan’s STMMs are mobilized on the basis of diplomatic considerations: Only a relatively small group of countries recognize the sovereignty of the Republic of China (Taiwan) [5,21]. Health aid to resource-limited countries has been proposed as a useful method to gain their friendship [22,23]. Taiwan has official diplomatic relations with only twenty-three nations, making this consideration perhaps different than it would be for people in many other countries. In an attempt to help both foster and cement diplomatic ties, the TaiwanICDF was established to represent Taiwan in overseas cooperation projects. Its funding comes from Taiwan’s Ministry of Foreign Affairs. In addition to providing medical aid, the TaiwanICDF aims to promote international relations between Taiwan and its developing partner nations. Providing economic assistance and foreign aid (such as grants, loans, and technical support) to diplomatic allies is part of Taiwan’s foreign policy [21]. On the other hand, the deployment of Taiwanese STMMs is based on Taiwan’s strategic interests and is designed to rally international support to break through diplomatic barriers set by the People’s Republic of China [21,23]. Therefore, Taiwan’s health aid is not only a humanitarian gesture but also a political consideration.

The number of health professionals who volunteer for STMMs has been rising quickly [24]. Although teamwork is an important factor in guiding the success of STMMs, very little research has been done to investigate the methods by which healthcare personnel are enrolled; and most articles on the subject are from self-published reports [1,7,9,12,19,25]. STMM members from multiple institutions may be unfamiliar with one another; managing such STMMs to ensure successful cooperation is therefore a challenge. In this study, we used a comparative survey to investigate the efficiency of STMMs. Our study demonstrated that STMMs in Central America and those in the South Pacific have similar efficiency, which suggests that clinical service provided by STMMs from multiple institutions has been as efficient as that of missions from a single institution.

In addition to the recruitment, we have disclosed two significant issues. First, language is an important skill in STMMs, with language barriers impairing communication between patients and healthcare providers [13]. Most mission members do not master Spanish. Our findings suggest that the development of a Spanish class focusing on medical terminology may be necessary to promote language proficiency among those who join STMMs to Central America. However, finding qualified teachers and teaching material, especially for the languages of remote tribes, is sometimes impossible outside the local setting. In addition, there is the question of how much language proficiency could practically be obtained in advance, especially for missions that may last only one or two weeks. Given the high level of satisfaction with the interpreters that were assigned to such missions, language did not appear to pose a substantial obstacle to STMMs that had translators available. Nonetheless, in cases where medical aid workers can speak additional languages, it would be beneficial to match, if possible, their service to destinations where such languages are spoken.

Second, our results indicate that most health professionals believed education can help local health workers to improve their knowledge and skills in health care. Although STMMs yield invaluable learning experiences, they fare less well in generating a meaningful, long-term impact on the provision of health care to local populations. It has been proposed that teaching is the most effective role played by STMMs, with capacity building through partnerships with local health workers being the most valuable [1,14,26,27]. Activities such as teacher training should be as important as individual encounters with patients. However, health personnel serving in Taiwan’s STMMs were often too busy to train local health workers. Furthermore, our study indicates that relatively little education was provided to local health workers by STMMs staffed from multiple institutions, especially in training for the administration of equipment (28%) and drugs (12%). This difference could be associated with several factors. First, there may be a lack of consensus among STMM members from multiple institutions about how to teach local health workers. Second, language is a significant barrier for missions to Central America. Third, there may be insufficient time to teach local health workers due to the heavier clinical loads that missions to Central America typically have. And fourth, the predominance of community-based missions to Central America and the Caribbean may limit educational opportunities for local health workers.

A couple of methodological issues should be cautiously interpreted in this study. First, inaccuracy may occur by reviewing the daily reports of STMMs. In this study, we used a questionnaire survey to reduce such possible bias. Second, our questionnaire is a self-estimated survey and did not explore the quality of health care. It may not reflect actual behavior in real-life situations. Further studies are needed to evaluate the impact of STMMs on the care outcome in recipient nations.

## Conclusions

This study has important implications for conducting a successful STMM. First, for the purpose of analysis, we drew a distinction between a STMM whose team came from a single hospital and one whose team came from multiple hospitals. The data demonstrate that multiple-hospital teams were as efficient in providing health care as those from just one hospital. However, missions recruited from multiple hospitals provided less educational training for local health workers. Second, our findings suggest proficiency in the native language of the target area is essential for the improvement of health care. Thus, development of classes in medical Spanish may be necessary for STMMs to Central America. Third, education for local healthcare workers is necessary to promote their skills in clinical care. STMMs should not only take care of patients in villages of underdeveloped countries but also improve the ability of local health care. In summary, this study has developed evidence-based policy recommendations to prepare for the provision of STMMs. We have identified some essential factors that could provide stakeholders and policymakers in building up STMMs. The findings in this study may provide further impetus for integrating evidence into the deployment of STMMs.

## Abbreviations

STMM: Short-term medical mission; TaiwanICDF: Taiwan International Cooperation and Development Fund; MOFA: Ministry of Foreign Affairs.

## Competing interests

All authors declare that they have no competing interests.

## Authors’ contributions

YWC, YHW, CFC, and CYY conceived of the analysis and participated in the design and coordination of the study, as well as in the acquisition and interpretation of the data. YWC and YHW conducted the literature review and drafted the initial manuscript. YWC, YHW, and CYY conducted the data analysis and editing. CFC, HYC, and MLL contributed to the overall drafting of the manuscript. All authors read and approved the final manuscript.

## Pre-publication history

The pre-publication history for this paper can be accessed here:

http://www.biomedcentral.com/1472-698X/12/37/prepub
